# A Simple Approach for Generating Random Aggregate Model of Concrete Based on Laguerre Tessellation and Its Application Analyses

**DOI:** 10.3390/ma13173896

**Published:** 2020-09-03

**Authors:** Yutai Guo, Jialong He, Hui Jiang, Yuande Zhou, Feng Jin, Chongmin Song

**Affiliations:** 1 State Key Laboratory of Hydroscience and Engineering, Tsinghua University, Beijing 100084, China; gyt18@mails.tsinghua.edu.cn (Y.G.); hjl16@mails.tsinghua.edu.cn (J.H.); hjiang@tsinghua.edu.cn (H.J.); jinfeng@tsinghua.edu.cn (F.J.); 2 School of Civil and Environmental Engineering, University of New South Wales, Sydney, NSW 2052, Australia; c.song@unsw.edu.au

**Keywords:** concrete meso-structure, random aggregate model, Laguerre tessellation, octree algorithm, scaled boundary finite element method

## Abstract

Generating random aggregate models (RAMs) plays a key role in the mesoscopic modelling of concrete-like composite materials. The arbitrary geometry, wide gradation, and high volume ratio of aggregates pose a great challenge for fast and efficient numerical construction of concrete meso-structures. This paper presents a simple strategy for generating RAMs of concrete based on Laguerre tessellation, which mainly consists of three steps: tessellation, geometric smoothing, and scaling. The computer-assisted design (CAD) file of RAMs obtained by the proposed approach can be directly adopted for the construction of random numerical concrete samples. Combined with the image-based octree meshing algorithm, the scaled boundary finite element method (SBFEM) was adopted for an automatic stress analysis of mass concrete samples, and a parametric study was conducted to investigate the meso-structural effects on concrete elasticity properties. The modelling results successfully reproduced the increasing trend of concrete elastic modulus with the grading of coarse aggregates in literature test data and demonstrate the effectiveness of the proposed strategy.

## 1. Introduction

Concrete is widely accepted as a composite material, and the main constituents at the meso-scale are commonly categorized into aggregate, mortar matrix, and interfacial transition zone. The internal heterogeneous structure contributes most to its complex behavior under a variety of loading conditions. Amongst the three-phase constituents, coarse aggregates normally occupy a large volume portion of a concrete mix, and their properties, such as size, shape, gradation, arrangement, and strength can influence the physical and mechanical properties of the final concrete batch to a great extent. Much attention has been given in literature to the effects of meso-structures on concrete properties, using theoretical, experimental, or numerical approaches. As an alternative to standard experimental techniques, numerical testing considering the meso-level particle structure [1] has become an important tool for examining concrete material properties. The interactions between aggregates and matrixes, as well as those between adjacent aggregates, have been frequently discussed in previous numerical investigations. Obviously, an appropriate representation of random aggregate distribution within the numerical concrete model forms the first basis of meso-scale investigations.

Furthermore, mass concrete containing maximum-size aggregates up to 150 mm are widely used in massive concrete structures, such as dam projects and offshore structures. In the dam engineering industry in China, dam concrete containing combined aggregate gradations of 5–20 mm, 20–40 mm, and 40–80 mm is classified as three-graded concrete, and termed as four-graded or fully graded concrete when another grade of aggregate of 80–150 mm is mixed together [2]. Normally these coarse aggregates occupy about one half of the total granular aggregates. It is also worthy to note that in the formation of rock-filled concrete (RFC), which has become a major type of construction material for dam structures in China [3,4], the maximum size of coarse aggregates can be even greater than 1000 mm. It is obvious that the existence of large or extremely large aggregates in concrete would demand the adoption of a sufficiently huge specimen for the determination of fundamental material behavior according to routine testing methods, and in turn would impose great challenges for the choice of testing machines. Overcoming these limitations is quite difficult, and wet-screened concrete is widely used instead for material testing by screening the aggregates larger than a certain size after mixing the components. However, it is obvious that wet-screening would alter the mixture proportions, and in turn affect the mechanical properties of the formed concrete [5]. Compared with laboratory material testing, the dimensions of aggregates pose no limitations on the application of meso-scale numerical simulations. However, due to the variable nature of aggregate size and shape, as well as the control of different packing densities, it is still quite challenging to develop a simple and direct approach for the buildup of random aggregate models (RAMs) within a mesoscopic concrete model. Quite a lot of attention has been given to such an issue in literature, and advances have been made in many aspects, not limited to the geometric generation of aggregates, placement or packing efficiency, and the penetration detecting of adjacent aggregates.

In the 1980s, Wittmann et al. [6], first used random polygons to simulate aggregates in the uniform mortar matrix, in which the number of polygon edges and inner angles were chosen as controllable variables, and the effects of meso-structures on crack propagation and failure processes were investigated. Since then, RAMs have been developed rapidly and numerous reports appeared in literature. Specifically, spherical particles [7], ellipsoidal aggregates [8], and polygonal and polyhedral aggregate models [9], have been adopted to simulate the meso-structure of concrete, spanning from two-dimensional (2D) to three-dimensional (3D). On the one hand, RAMs help to deepen the description and understanding of the meso-structure of concrete and the macro–meso coupling mechanism, but on the other hand, the present approaches for generating RAMs still suffer from relatively low computational efficiency and the volume content of generated aggregates can barely satisfy the aggregate gradation requirements of fully graded concrete and RFC. From the perspective of whether to directly consider the penetration of adjacent aggregates, the approaches for RAM buildup can be mainly divided into two categories. For those of the first category, a step for checking potential penetration between adjacent aggregates is necessary in the procedures, such as the method by Wang [10] for simulating arbitrary shape aggregates in two dimensions, and the convex extension method by Ma et al. [11] to construct convex polyhedral aggregates. For those of the other category, numerical aggregates are generated in a bounding box, such as the layering disposition method [12], which customizes a cuboid bounding box for each aggregate and then places the boxes within the specimen space in a random order. The incorporation of the bounding boxes during aggregate packing can ensure that there is no penetration between adjacent aggregates. The advantage of the layering disposition method is time-saving, but the maximum achievable aggregate content is limited and comparatively low. Recently, Voronoi grains were chosen as bounding boxes, such as in the Packing-3D program by Mollon and Zhao [13], by which the upper limit of the aggregate content can be raised to a notable extent. The Packing-3D program has been widely used in discrete element modelling of granular materials, showing a higher degree of flexibility in the consideration of aggregate shape parameters such as aspect ratio, roundness, and sphericity. The authors tried to extend this program for generating RAMs for fully graded concrete, and found it incapable of simulating the dense arrangement of coarse aggregates in dam concrete practice.

For continuum meso-scale numerical investigation into concrete materials, finite element discretization is another challenging task following the generation of numerical aggregates. Owing to the randomness and irregularity of the polyhedron aggregates, convex or concave, constructing hexahedral mesh elements for representing concrete meso-structures, which are normally preferred in most circumstances, still remains a great challenge. When an accurate characterization of the outer surfaces of aggregate solids is demanded, the aligned meshing approach based on tetrahedral elements become a viable choice [14]. On the other hand, the mapping mesh method [15] is more frequently adopted due to its simplicity, of which the main steps are described as follows. Firstly, a regular tetrahedral or hexahedral elements-based grid is generated for the targeted concrete specimen. Secondly, the material property definition of each background element is determined by mapping its coordinates of centroid with the occupied volume of aggregates, and those with centroids outside all aggregates are defined as belonging to the matrix component. When the grid size is fine enough, it is deemed that the mapping mesh approach can appropriately characterize the complex meso-structures of the concrete. It is worthy to note that for aggregates of complex topology, both the aligned meshing approach and the mapping mesh method, would require considerable user interactions. In the 1990s, Song and Wolf [16], proposed the scaled boundary finite element method (SBFEM), whose semi-analytical characteristics make it absorb the advantages of the finite element method and the boundary element method, so it has been widely used in solving elastic statics problems [17], and elastic dynamics problems [18]. In recent years, SBFEM has been developed continuously by constructing polyhedral elements with an arbitrary number of faces and nodes, which makes it more flexible. Saputra et al. [19], developed a SBFEM program combining quadtree (2D) and octree (3D) algorithms for automatic image-based meshing and stress analysis, which presents notable advantages in characterizing complex geometric structures. Moreover, Xing et al. put forward a node-to-node (NTN) scheme for modeling contact problems in two-dimensional [20] and three-dimensional [21] models, using SBFEM.

Following the second category of approach for generating RAMs mentioned above, this paper presents a simple and efficient method to generate three-dimensional RAMs. Specifically, Laguerre tessellation is considered as the initial bounding boxes for aggregates, and the actual grading requirements can be satisfied by a proposed grading adjustment strategy. Furthermore, based on the CAD file of RAMs, the SBFEM program combined with the octree algorithm is used to mesh irregular-shaped aggregates and the matrix component automatically, and the factors affecting elastic constants of large aggregate concrete are discussed from a mesoscopic point of view.

## 2. Strategy for Generating 3D RAMs

**Figure 1:**
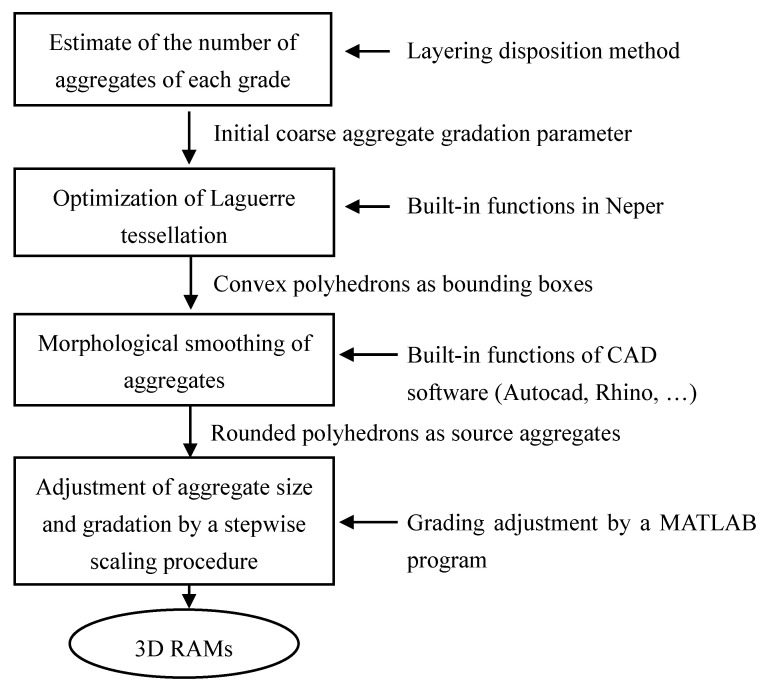
Flowchart of generating 3D random aggregate models (RAMs) of concrete.

Borrowing the ideas from the layering disposition method [12] and the Packing-3D program [13], a new strategy for generating 3D RAMs of mass concrete is proposed in this study. The main steps are described as follows. Firstly, Laguerre tessellations are generated within the specimen space according to the prescribed aggregate size and gradation requirements, and the generated cells are adopted for the definition of the initial bounding boxes of aggregates. Secondly, to overcome the unnecessary parallelism of adjacent tessellation faces, a geometrical smoothing procedure is conducted and each generated cell takes a rounded outer shape. Meanwhile, this procedure also creates a tiny gap between adjacent cells. Thirdly, the cells are categorized into groups according to the grading of coarse aggregates, and a stepwise scaling treatment is conducted on each group individually in order of decreasing size, and a geometry model of RAMs satisfying the requirements of aggregate gradation and volume ratio is obtained. The proposed strategy avoids the time-consuming penetration detections between aggregates, and retains the diversity of complex aggregate shapes. The flowchart of the strategy for generating 3D RAMs is summarized in Figure 1. More details of the generation procedures are given in the following sections. Section 2.1 presents the principles of Laguerre tessellation generation process, considering the desired grain size distribution. Section 2.2 details the aggregate gradation adjustment algorithm and presents some numerical instances of 3D RAMs within a 450 mm cubic sample of fully graded concrete.

### 2.1. Construction of Laguerre Tessellation

The construction of Voronoi tessellation is commonly used to represent the polycrystalline structure and can effectively divide the specimen space into a collection of polyhedrons without overlaps and gaps by putting random seeds inside and generating lines and faces around each seed. These entities are defined as influence zones of a particular set of seeds. Recently, the development of Laguerre tessellation, a generalized version of Voronoi tessellation, made it more feasible to control morphological properties of these polyhedrons, such as cell size distribution, sphericity, and aspect ratio, by optimization of the objective function. Fundamentals of Laguerre tessellation are described as follows, and more details can be found in [22].

Given a spatial domain $D\in R^{N}$ and *n* uncorrelated seeds randomly distributed within D,$\left\{\;S_{i}\left(x_{i},w_{i}\right),i=1,\ldots ,n\;\right\}$, where *X_i_* indicates seed coordinates and *W_i_* denotes the nonnegative weight coefficient at each seed point. The Laguerre element *C_i_* corresponding to each seed can be expressed as:

(1) $$C_{i}=\{P\left(x\right)\in D\;|\;d_{L}(P,S_{i})<d_{L}\left(P,S_{j}\right)\;\forall j\neq i\}$$

(2) $$d_{L}\left(P,S_{K}\right)=d_{E}{\left(P,S_{K}\right)}^{2}-w_{k}$$

where *d_L_* is defined as the power distance and *d_E_* is the Euclidean distance.

The process of adjusting seed properties to obtain the desired cell properties, herein mainly of grain size, shape, and gradations, can be transformed into an optimization problem of the objective function. The optimization variables include the seed coordinates and the square root of weight coefficient, and the number of optimization variables is *4N* in 3D space. A metric function is introduced by comparing the distribution *X(x)* of samples during the iteration with the specified probability distribution *F(x)* as:

(3) $$\Phi =\int_{-\infty }^{+\infty }\frac{{\left[X\left(x\right)-F\left(x\right)\right]}^{2}}{F\left(x\right)\left[1-F\left(x\right)\right]}dx$$

where *X(x)* is a step function whose expression is:

(4) $$X\left(x\right)=\frac{1}{N}\sum_{i=1}^{N}H\left(x-x_{i}\right)$$

(5) $$H\left(k\right)=\{\begin{array}{cc} 0 & if\;k<0\\ 1 & if\;k\geq 0 \end{array}$$

Numerical integration is adopted to calculate *Φ* and a minimum absolute error of the objective function is used to terminate optimization. In this paper, the Neper program by Quey and his colleagues [22] was adopted for tessellation, in which the cell size distribution and the number of cells are specified as input parameters. For simplicity, the cell size is defined as the diameter of a sphere of equal volume, and combined aggregate gradations can be achieved by the combination of different normal distributions. The number of cells, another input parameter, indicating the number of aggregates at each grading, is estimated based on the layering disposition method [12], of which the main steps are as follows: (1) generate aggregates according to the prescribed size and fraction proportions, wrap each of them with a cuboid box, and arrange them in random order in a list; (2) locate each cuboid within the specimen space from bottom to top in order and at the lowest part of the current layer; (3) adjust the distance of bounding cuboids for prescribed packing density; (4) terminate the disposition process when all aggregates are arranged within the specimen space, and restart if there are remaining aggregates.

As the generated tessellations would occupy the complete specimen space, the mean cell size of each grading would reasonably be greater than the actual value, and the subsequent geometric smoothing and scaling adjustment are required to achieve the designed aggregate gradation of mass concrete.

### 2.2. Geometric Smoothing and Aggregate Gradation Adjustment

#### 2.2.1. Morphological Smoothing of Aggregates

One can note that there are no overlaps or gaps between adjacent polyhedrons in the tessellation model, and the union of all polyhedrons occupies the whole space of a given specimen, indicating an aggregate ratio of 100%. In order to meet the requirements of 40%–70% aggregates volume content within mass concrete, and avoid the complex interparticle penetration detection process, the random convex polyhedrons generated by the Neper program in Section 2.1 are adopted as the initial bounding boxes of coarse aggregates. One simple method to lower the volume of aggregates is to conduct a geometric smoothing on all the polyhedrons using the built-in functions in many CAD software programs, such as the Meshsmooth command in Auto-CAD^®^ (Autodesk, Inc., Mill Valley, CA, USA). The roundness of each aggregate cell can be increased by raising the smoothness levels, or vice versa. It is worth noting that such a smoothing procedure can also remove the unnecessary parallelism between original common facets of adjacent aggregates and obtain more realistic aggregate shapes, at the cost of increasing the number of facets surrounding each cell.

**Figure 2:**
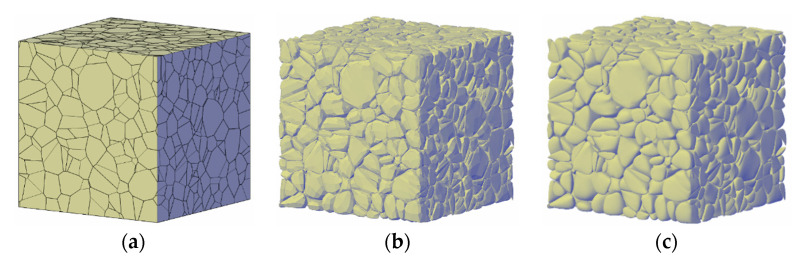
Initial Laguerre tessellation and polyhedrons after geometric smoothing in a cubic sample. (**a**) Initial Laguerre tessellation (smoothness level = 0), (**b**)smoothness level = 1, and (**c**) smoothness level = 3.

Figure 2 presents typical results of the Laguerre tessellation of a cubic sample and the rounded polyhedrons after geometric smoothing using different parameters of smoothness level. The figure results clearly demonstrate the smoothing effect and the potential of controlling the roundness of aggregates.

#### 2.2.2. Grading Adjustment Algorithm and Implementation

After the above geometry smoothing procedure, all the rounded aggregates are categorized into groups and numbered according to their characteristic size in an ascending order as follows:

(6) $$Group_{i}=\{aggregate_{j}\;|\;d_{i,min}\;\leq d_{aggregate_{j}}\;\leq d_{i,max}\}$$

Where *Group_i_* records the aggregates currently belonging to *i*-th group, and *d_i,min_* and *d_i,max_* represent the minimum and maximum aggregate size of the *i*-th group, respectively. Take the three-graded concrete for instance, three groups are to be defined corresponding to small stones (5–20 mm), medium stones (20–40 mm), and large stones (40–80 mm). For those aggregates larger than the maximum size of all groups, they are categorized into the coarsest group. The cells after geometric smoothing that are smaller than lower bound of coarse aggregates are removed from the RAMs.

In order to meet the size requirements, a geometric downscaling is applied with reference to the centroid of each aggregate and the dimensions of aggregates within each group can be adjusted. To fulfill the gradation or the ratio requirements of each aggregate group, a step-wise scaling procedure for the categorized aggregate groups is proposed and given below.

With a scaling factor input as *S*%, the characteristic size and volume of an aggregate after scaling are determined as follows:

(7) $$d_{new}=d*S\%$$

(8) $$V_{new}=V*S{\%}^{3}$$

Similar to the arrangement of nested sieves in order of decreasing size from top to bottom during a sieve analysis test, the proposed scaling procedure is to be conducted in a descending order, that is, from large-sized group to small-sized one. It should be noted that the aggregates belonging to the large-sized group can be downgraded into the small-sized group after a geometric reduction, hence the grouping information of all aggregates needs to be updated after a scaling treatment.

For the *i*-th group, the initial scaling factor *S*_0_% is determined by

(9) $$S_{0}\%=1-\sqrt[{3}]{\frac{V_{i,pres}}{V_{i,sum}}}$$

in which *V_i,pres_* is the prescribed total volume of the *i*-th group aggregates by the design gradation, and *V_i,sum_* is the volume value at the present stage.

With the initial scaling factor *S*_0_% given, scaling operations are looped by writing a scripting program in MATLAB with a small decrease rate, 1‰ suitable for most circumstances in practice, until the total volume of all aggregates that still belong to the *i*-th group is equal to *V_i,pres_* A similar scaling process is followed for (*i*-1)-th group until all groups are scaled and the prescribed gradation is satisfied.

#### 2.2.3. Examples of Generating 3D RAMs of Mass Concrete

The generation procedures for 3D RAMs within a 450 mm-sized cubic fully graded concrete specimen is given in this section. The coarse aggregates are normally divided into four groups: 5–20 mm, 20–40 mm, 40–80 mm, and 80–150 mm. To lower the computational burden, the aggregates of the smallest-sized group, 5–20 mm, are neglected in our RAMs model, which are to be equivalently treated as the component of matrix.

The aggregate gradation of fully graded concrete in the testing study by Deng et al. [23] was chosen (Table 1), and a density of 2.7 × 10^−6^ kg/mm^3^ was adopted for the aggregate rock. The design volume ratio of coarse aggregates in Table 1 is 48.6%.

Firstly, using the layering disposition method [12] provided an estimate of approximately 1500 coarse aggregates in total within the 450 mm-sized numerical specimen, and the number of aggregates within each group was estimated. A grouping instance of all aggregates consisted of 1230 medium stones (20–40 mm), 230 large stones (40–80 mm), and 40 extra-large stones (80–150 mm). Secondly, given the ratio of aggregate number in all groups, random Laguerre tessellations could be obtained for the cubic specimen by the Neper program, with an assumption that the aggregate size within each group conforms to the normal distribution. It was appropriate to raise the average cell size of each group by 10%–20% considering the Laguerre tessellation of the complete sample space and subsequent scaling procedure. Thirdly, a geometric smoothing was carried out on all the generated cells using Auto-CAD software. Finally, a step-wise adjustment of the ratio of aggregate number was conducted for each group and the corresponding scaling factors determined by the algorithm in Section 2.2.2 are given in Table 2.

**Figure 3:**
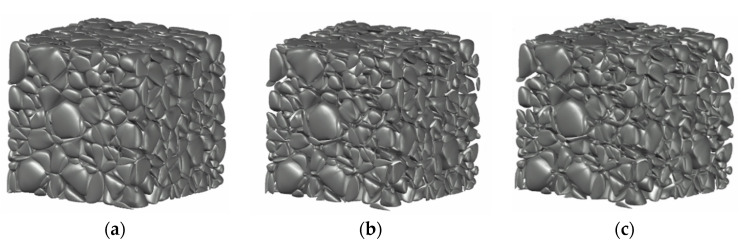
RAM distributions of cubic fully graded concrete samples during scaling procedures. (**a**) After scaling of the 80–150 mm group, (**b**) after scaling of the 40–80 mm group, and (**c**) after scaling of the 20–40 mm group.

**Figure 4:**
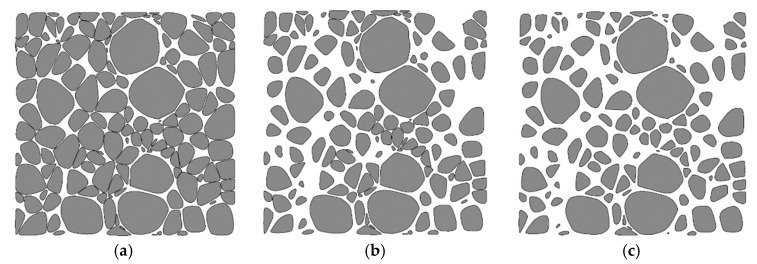
Aggregate distribution development at a slice during the scaling procedures. (**a**) After scaling of the 80–150 mm group, (**b**) after scaling of the 40–80 mm group, and (**c**) after scaling of the 20–40 mm group.

It can be seen that after morphological smoothing treatment, the ratio of aggregate number in each group was 1018:432:42, which was then successively adjusted by the scaling treatment of each group following a descending order of the size. After the final scaling treatment on the 20–40 mm group, the volume ratio of aggregates became 1.11:1.66:1.66, the same as that required in the testing study [23]. Figure 3 presents typical 3D RAM distributions within a cubic sample during the stepwise scaling procedure, and a slice illustration of the variation of RAMs at one-third of the sample height is also shown in Figure 4. Both the final 3D RAM model in Figure 3 and the slice distribution of aggregates in Figure 4 clearly demonstrate the random distribution of aggregate, both in size and in shape, within the specimen space, and the effectiveness of geometric smoothing and scaling procedures on the adjustment of aggregate size and gradation is also proven.

## 3. Mesoscopic Investigation into Elasticity Properties of Mass Concrete

### 3.1. Automatic Image-Based Stress Analysis Using SBFEM

The above strategy simplifies the task of generating various RAM structures of concrete to a notable extent, and provides a high flexibility in the adjustment of aggregate size and gradation, as well as the volume ratio of aggregates. This section adopts the strategy for generating RAMs of typical mass concrete and presents a mesoscopic investigation into the effects of meso-structures on the overall elasticity properties. Particularly, the three-graded and fully graded concrete materials, featuring a broad range of aggregate size, were selected in this study. It is worthy to note that the present approach can be smoothly extended for the analysis of RFC, which is characterized by dense packing of extremely large aggregates over 1000 mm in size.

As mentioned above, the complex shapes of aggregates would complicate the discretization process using structured or unstructured meshing techniques commonly chosen in the establishment of an FE model, and ensuring the mesh quality for the meso-structure is not an easy task. On the other hand, an automatic image-based octree meshing approach was developed by Song and his co-workers [19,24], which is capable of converting 3D image data into high-quality scaled boundary polyhedral meshes, and its high flexibility of element shapes eliminates the hanging node issue in FEM analysis and significantly benefits an efficient transition between elements of different sizes. In this section, the SBFEM code by Song [24] was adopted to conduct the following mesoscopic analyses.

**Figure 5:**
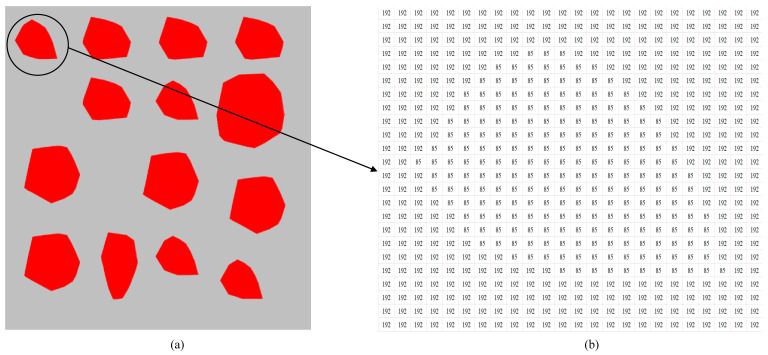
Typical slice of RAM of concrete (**a**) and the corresponding color matrix (**b**).

With the CAD model of the RAMs generated, it is quite simple and direct to provide a stack of 2D slices describing the geometrical features of all coarse aggregates within a concrete sample. The residual domain, excluding the aggregates, is defined as the matrix component. It is worthy to note that when necessary, it is straightforward to incorporate the role of the interfacial transition zone by simple bolding of the boundary lines of all aggregates during the image-based generation of octree mesh. For completeness, the elementary procedures for the generation of octree polyhedral mesh and the subsequent SBFEM analysis for a cubic concrete specimen are summarized as follows, whilst more details and the theory of SBFEM can be found in Song [24]:

1. Generate a stack of 2D slices from the CAD file of 3D RAM, and disintegrate the aggregate component from the matrix by the assignment of contrasting color intensities. Figure 5 presents a typical 2D slice of numerical concrete specimen and its corresponding color matrix, in which the red zone defines the distribution of aggregates, and the grey zone indicates the surrounding matrix component, with contrasting mean RGB values of 85 and 192, respectively.
2. Divide the 3D image assembled from the stack of 2D slices into cubic cells, and construct a cubic image matrix, I_bound_ to store the colors of all voxels, whose size must be integer power of 2, that is, 2^n^ × 2^n^ × 2^n^.
3. Conduct the octree decomposition of I_bound_ according to the recursive algorithm by Saputra et al. [19], and the cells forming the numerical specimen must satisfy the prescribed criterion of homogeneity or reach the minimum cell size *s*_min_.
4. Assign material properties (aggregate or matrix) to each octree cell according to its representative color by averaging the color intensity values of internal voxels.
5. Each octree cell is modelled as a scaled boundary polyhedral element and assembled into the system balance equation in the context of SBFEM. The nodal displacements can be solved, and in turn the stress distribution within the specimen is determined.

**Figure 6:**
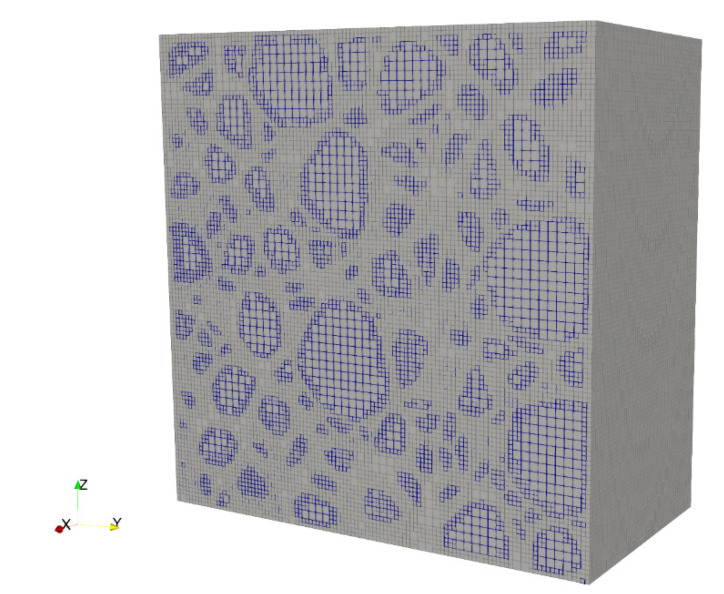
Balanced octree meshing of a typical fully graded concrete sample.

For all the numerical concrete samples in this study, the maximum and minimum values of octree edge length were chosen as *s_max_* = 8 mm and *s_min_* = 4 mm, and the maximum edge length ratio between adjacent cells was defined as not greater than 2:1, such that a balanced octree decomposition, as proposed by Saputra et al. [19], could be obtained, which effectively limits the number of potential octree cell patterns in the numerical samples and greatly lowers the computation cost. Figure 6 illustrates a sectional distribution of polyhedral elements considering the complex shape of aggregates (indicated by blue boundary elements) surrounded by the matrix component (shown as elements with light grey boundaries). It was found that a refined cell grid was effectively defined along the peripheral area of aggregates, which is advantageous for approaching the actual distribution of aggregates and simulating the matrix–aggregate interaction. Linear elastic constitutive models were adopted for both aggregates and matrix components, and a vertical support boundary condition was applied at the bottom of each specimen.

### 3.2. Mesoscopic Analysis of Elasticity Properties of Mass Concrete

#### 3.2.1. Effects of Coarse Aggregate Gradations

**Figure 7:**
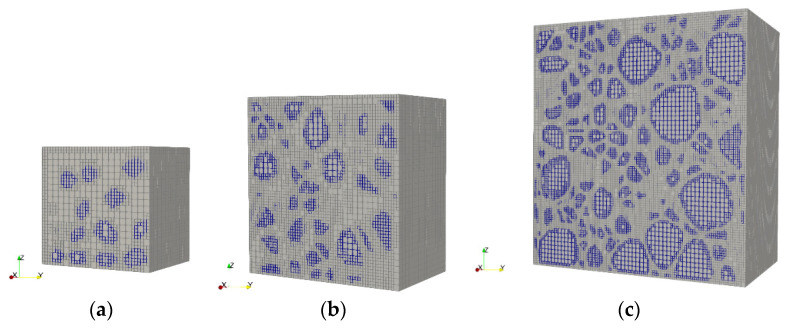
Typical octree mesh of aggregates and matrix of wet-screened (**a**), three-graded (**b**), and fully graded (**c**) concrete samples.

As mentioned above, fundamental testing of concrete material demands that the specimen size is sufficiently large, as compared to the maximum aggregate size, which would bring up difficulties in the preparations of testing samples and the selections of testing machine for mass concrete with extra-large aggregates. Hence, the wet-screening method is commonly utilized in dam engineering practice by sieving out the over-sized aggregates of the fresh concrete, and the reduction of maximum aggregate size makes it feasible using small samples and the usual laboratory testing machines. Obviously, such a treatment would significantly change the size and gradation properties of aggregates in the samples as compared with the mass concrete in practice. To illustrate the effect of such a sieving procedure on the elasticity properties of concrete, three sets of UC testing specimens were generated and analyzed using the above automatic image-based approach by the SBFEM, including three-graded and fully graded dam concrete, as well as the wet-screened concrete by sieving out aggregates greater than 40 mm from the three-graded concrete batch. Five cubic specimens with random RAMs were considered in each set using the above strategy in Section 2, and typical instances are shown in Figure 7. The overall elastic modulus of each specimen was determined from the UC analysis results and the effect of aggregation gradation is discussed as follows.

The proportions of coarse aggregates within three-graded and fully graded numerical concrete specimens were selected according to relevant testing study [23,25], in which a series of laboratory tests have been carried out on the performance of concrete materials with variable aggregate gradations under uniaxial and multiaxial loading conditions. A summary of the aggregate proportions is given in Table 3 and above in Table 1, respectively. The smallest grade of aggregate, 5–20 mm in size, was equivalently treated as matrix component and not explicitly simulated. One can note from Figure 7 that an edge length of 250 mm (450 mm) was chosen for the three-graded (fully graded) concrete specimen, and a size of 150 mm was selected for the wet-screened specimen, all of which fulfilled the requirement of characteristic sample size three times greater than the maximum aggregate size.

The elastic parameters describing the meso-level constituents within each specimen, aggregates, and matrix are given in Table 4, which both lies within the commonly used range of elastic constants in a meso-structural analysis of concrete material. From the UC modelling results of each set of specimens, a summary of the elastic constants of wet-screened, three-graded, and fully graded samples are given in Table 5.

The results in Table 5 demonstrate that the adjustment of coarse aggregate gradations would notably influence the overall elasticity properties of formed concrete, and an increase trend is shown in elastic modulus with the grade of the maximum aggregate fraction. It was also found that compared with the results from wet-screened specimens with coarse aggregate sieved out, the elastic modulus of three-graded and fully graded concrete materials presented an increase by 11% and 22%, respectively. On the other hand, only a minor difference was shown in the results of Poisson’s ratio determined from the meso-scale analyses of the three types of concrete. Similar results can be found in literature on the change of elastic modulus in relation to the coarse aggregate gradations. Table 6 presents a summary of the elastic modulus distributions of three-graded and fully graded concrete from experimental studies in literature. It can be observed that with the modulus of wet-screened concrete taken as the reference value, the calculated ratio of elastic modulus of three-graded and fully grade concrete in this study are in agreement with the test results, which on the other hand may also approve the proposed strategy for generating RAMs of mass concrete and the suitability of automatic image-based analysis by SBFEM.

#### 3.2.2. Effects of Size-Reduced Sampling

For mass concrete with extremely large aggregates in practice, such as the above mentioned RFC with aggregates that may be of 1000 mm in size, the routine practice of taking specimen size two or three times greater than the maximum aggregate size poses a huge challenge to the loading capabilities of testing machines. A common compromise is to core or cut smaller samples for testing, and it is no wonder that the testing results of fundamental parameters, such as elastic modulus and uniaxial compressive strength would be influenced by such a size reduction. This section takes the above numerical sample (Figure 6) of fully graded concrete as the source concrete batch, and five cubic samples of 225 mm in size were randomly manufactured by cutting. Meanwhile, another five cubic samples of the same size were generated by the proposed strategy in Section 2 according to the grading requirements of fully graded concrete, that is, at a design level of 48.6% coarse aggregate content in volume and a volume ratio of aggregates in each group at 1.38:2.08:2.08 (×10^6^ mm^3^). A comparison study was conducted by UC modelling of all these ten specimens, and the effects of the size-reduced sampling approach in practice was investigated.

From the simulation results of the two sets of specimens, a comparison of the elastic moduli is shown in Table 7 and Table 8. The mean values of elasticity constants from the two sets of specimens are basically the same. It is worthy to note that the results of samples meeting the grading requirements in Table 7 presented relatively minor difference, whilst comparatively larger scatter was shown by the results of the other set of samples randomly cut from a larger batch in Table 8. It is also found that both the volume and gradation of coarse aggregates of the five randomly cut samples differed much from the design value, which caused the notably scattered results of elastic constants in Table 8.

#### 3.2.3. Effects of 2D Simplification

To lower the computation complexity and cost, 2D simplification is commonly adopted for meso-scale modelling of the complex behavior of concrete. It is obvious that the non-uniform spatial distribution of aggregates within actual concrete material cannot be well represented by plane modelling of typical sections. It is interesting to raise the question of whether a 2D model with coarse aggregate fulfilling the grading requirements is representative of the overall elastic behavior of concrete, or representative of the variations of aggregate distributions along slices at different portions and their effect on the evaluation of elastic properties. In this section, we generated four 450 mm-sized cubic samples of fully graded concrete to simulate the concrete batches, using a volume ratio of 52% of coarse aggregates larger than 20 mm. A thin layer of 5% of the specimen size along the outer surface of each batch was removed to minimize the boundary effects. Without loss of generality, 400 slices parallel to one surface at an interval of 1 mm were generated from the remaining cubic sample. The aggregate content of each slice was calculated and UC modelling was conducted to determine the corresponding elastic modulus. Both plane stress and plane strain analyses were adopted and the calculation results are summarized in Table 9.

The results in Table 9 demonstrate that with a sufficient number of slices, the mean values of aggregate contents of all slice models within the four source specimen fell in a range of 50.7%–51.4%, which is basically consistent with the prescribed aggregate content of 52% for each 3D specimen. Nonetheless, a finite range of aggregate ratios, roughly in the range of 43%–58%, were monitored from the slices of all specimens using the RAMs generated by the above strategy, which are reasonably attributed to the 3D random distribution of RAMs. The UC analysis results of all the slices based on plane strain (plane stress) assumption provide an estimate of the overall elastic modulus in the range of 32.9–36.1 GPa (31.9–35.1 GPa), and the plane strain modelling results present a relatively higher modulus as compared to those from plane stress analyses. Similar to the above findings from 3D modelling, a larger coarse aggregate ratio in the slice would give rise to an increase of the elastic modulus, and the 2D specimens of a mean aggregate ratio of about 51% give an acceptable estimate of the elastic modulus as the 3D modelling results, particularly, when a plane stress assumption is adopted. It is also worthy to note that 2D simplification is questionable in nonlinear analysis of massive concrete structures.

## 4. Conclusions

This paper presents a simple strategy for generating 3D RAMs of concrete materials, and its combination with SBFFEM and the image-based octree meshing technique allows a simple and efficient investigation into the meso-structural effects on the macro-scale mechanical behaviors of concrete. The proposed strategy firstly utilizes Laguerre tessellation for the definition of bounding boxes for the aggregates according to their size and gradation requirements, and then these tessellation cells are subjected to controlled geometric smoothing in CAD program, which can remove the unnecessary parallelism of common facets of adjacent cells and introduce a finite gap between them. The RAM generation procedure is finalized by a stepwise scaling procedure, which can efficiently tune the size and in turn the gradation of cells exactly the same as the prescribed aggregate fraction ratio. Numerical examples were given, which demonstrated the effectiveness of the proposed strategy for generating three-graded and fully graded mass concrete samples. The aggregate volume ratio can be larger than 50% and even higher. With the CAD model of the RAM generated, it is quite direct to conduct automatic image-based stress analysis using SBFEM considering the meso-level aggregate structure. Numerical studies were subsequently conducted, aiming to investigate the effects of aggregate gradation, size-reduced sampling, and 2D simplification on the evaluation of overall elasticity properties of mass concrete. The simulation results reproduced the increasing trend of the elastic modulus with the grading of maximum aggregate in literature test reports, and the simulations based on size-reduced sampling and 2D simplification can introduce relatively large scatter in results owing to the randomness of 3D aggregate distribution within a concrete specimen.

The paper provides a simple and efficient strategy for the construction of random numerical samples of concrete, considering high aggregate content and broad range of gradation. With additional work of compaction simulation, it can be extended for the simulation of dense packing of extremely large aggregates in RFC, of which the size may be greater than 1000 mm and the aggregate volume ratio can be as large as 60%. Moreover, the automatic image-based SBFEM modelling is proven to be appropriate for meso-level simulation of the elasticity properties of mass concrete, and can be extended for nonlinear damage and failure analysis under this framework.

## Figures and Tables

**Table 1:** Aggregate components of fully graded concrete

	Size Grouping of Coarse Aggregates/mm		
	20–40	40–80	80–150
Volume (×10^7^ mm^3^)	1.11	1.66	1.66
Volume ratio (%)	12.15	18.22	18.22

**Table 2:** Scaling factors and gradation changes during geometric smoothing and scaling procedures.

Grading Adjustment	Geometric Smoothing of Aggregates	Scaling of Aggregates		
		80–150 mm	40–80 mm	20–40 mm
Factor *S*%		4.7%	20.15%	12.0%
Ratio of aggregate number in each group *	1018:438:42	1018:444:36	1163:299:36	1163:299:36
Volume ratio of aggregates in each group (×10^7^/mm^3^) *	1.26:3.86:2.1	1.26:4.01:1.66	1.64:1.66:1.66	1.11:1.66:1.66

* The ratio is given in an ascending order of aggregate size in each group.

**Table 3:** Aggregate proportions of three-graded concrete [25].

Coarse Aggregate Size (mm)	20–40	40–80
Mass (10^−9^ kg/mm^3^)	407.1	542.8

**Table 4:** Elastic parameters of meso-level constituents within concrete specimen.

	Elastic Modulus (GPa)	Poisson’s Ratio	Density (×10^−6^ kg/mm^3^)
Aggregate	50	0.15	2.7
Matrix	25	0.18	2.1

**Table 5:** Comparison of calculated elastic constants of concrete samples of different aggregate gradations.

Type	Wet-Screened	Three-Graded	Fully Graded
Specimen size (mm)	150 × 150 × 150	250 × 250 × 250	450 × 450 × 450
Number of coarse aggregates (>20 mm)	62	352	1625
Aggregate volume ratio (%)	15.1	35.2	48.6
Elastic modulus (GPa) *	27.37 ± 0.033	30.43 ± 0.014	33.41 ± 0.035
Poisson′s ratio *	0.169 ± 0.0012	0.168 ± 0.00068	0.163 ± 0.0023

* Mean value and standard deviation of the results from all five samples within each set are given.

**Table 6:** A comparison of the elastic modulus of mass concrete with reference to the wet-screened concrete from test and numerical studies.

	Wet-Screened (GPa)	Three-Graded (GPa)	Fully Graded (GPa)	Three-Graded/Wet-Screened	Fully Graded/Wet-Screened
Serra et al. [5,26]	27.9	29.7	33.5	1.06	1.20
	25.6	28.4	32.5	1.11	1.14
Vilardell et al. [27]	35.1	-	43	-	1.23
Li & Yang, [28]	-	-	-	-	1.15
Yang, [29,30]	33.45	37.05	-	1.11	1.22
Qu et al. [25]	29.6	32.3	-	1.09	-
Zhou et al. [31]	-	-	-	-	1.18
Tang et al. [32]	-	-	-	-	1.13
Dong et al. [33]	30.05	-	34.2	-	1.14
Zhu, [34]	27.16	-	31.76	-	1.17
Li, [35]	36.85	-	44	-	1.19
Yang & Li, [36]	29.93	32.675	-	1.09	-
Present study	27.37	30.43	33.41	1.11	1.22

Note: “-” in the table indicates that the corresponding item is not available from the literature report, whilst the given values represent the average value determined from corresponding testing results or given by the present numerical study.

**Table 7:** A summary of aggregate parameters and calculated elastic constants of samples generated with gradation requirements fulfilled.

Sample No.	Number of Coarse Aggregates	Elastic Modulus (GPa)	Poisson’s Ratio
1	200	33.2	0.16
2	200	33.2	0.16
3	210	33.4	0.16
4	190	33.2	0.17
5	195	33.2	0.16
Mean and standard deviation	199 ± 7	33.2 ± 0.08	0.16 ± 0.003

**Table 8:** A summary of aggregate parameters and calculated elastic constants of samples randomly cut from the same concrete batch.

Sample	Number of Aggregates	Volume Ratio of Aggregates (%)	Elastic Modulus (GPa)	Poisson’s Ratio	Volume Ratio of Aggregates in Each Group (×10^6^ mm^3^)
1	229	45.42	32.6	0.16	1.67:2.6:0.91
2	253	49.43	33.5	0.17	1.55:2.36:1.71
3	209	51.4	33.9	0.17	1.28:2.4:2.18
4	251	44.84	32.3	0.16	1.83:2.44:0.84
5	299	51.87	33.9	0.16	1.9:2.39:1.62
Mean and standard deviation	248 ± 30	48.59 ± 2.95	33.2 ± 0.67	0.16 ± 0.004	

**Table 9:** Comparison of coarse aggregate fractions in 2D slices and statistical values of calculated elastic moduli.

Source Concrete Sample	Statistical Values of Coarse Aggregate Volume Ratio in All 2D Slices (%)			Statistical Values of Calculated Elastic Modulus of Each 2D Slice Model (GPa)			
				Plane Strain		Plane Stress	
	Mean	Range *	Standard Deviation	Mean	Range	Mean	Range
#1	51.1	(47.0–58.2)	2.45	34.4	(33.6–36.1)	33.4	(32.6–35.1)
#2	50.7	(43.6–57.8)	3.01	34.3	(32.9–36.0)	33.3	(31.9–35.0)
#3	51.3	(44.7–56.1)	2.68	34.4	(33.1–35.7)	33.4	(32.1–34.7)
#4	51.4	(44.3–57.7)	3.73	34.3	(33.0–35.8)	33.3	(32.0–34.8)

* The two numbers within the bracket represent the lower bound (left) and upper bound (right) values of all slices from the same sample.
